# A novel index to aid in prioritizing habitats for site‐based conservation

**DOI:** 10.1002/ece3.8762

**Published:** 2022-03-25

**Authors:** Anthony Basooma, Herbert Nakiyende, Mark Olokotum, John S. Balirwa, Winnie Nkalubo, Laban Musinguzi, Vianny Natugonza

**Affiliations:** ^1^ 187216 Capture Fisheries & Biodiversity Conservation Programme National Fisheries Resources Research Institute Jinja Uganda; ^2^ Maritime Institute Busitema University Tororo Uganda

**Keywords:** Africa, biodiversity loss, GBIF, IUCN, multiple stressors

## Abstract

Funding biodiversity conservation strategies are usually minimal, thus prioritizing habitats at high risk should be conducted. We developed and tested a conservation priority index (CPI) that ranks habitats to aid in prioritizing them for conservation. We tested the index using 1897 fish species from 273 African inland lakes and 34 countries. In the index, lake surface area, rarity, and their International Union for Conservation of Nature (IUCN) Red List status were incorporated. We retrieved data from the Global Biodiversity Information Facility (GBIF) and IUCN data repositories. Lake Nyasa had the highest species richness (424), followed by Tanganyika (391), Nokoué (246), Victoria (216), and Ahémé (216). However, lakes Otjikoto and Giunas had the highest CPI of 137.2 and 52.1, respectively. Lakes were grouped into high priority (CPI > 0.5; *n* = 56) and low priority (CPI < 0.5; *n* = 217). The median surface area between priority classes was significantly different (W = 11,768, *p* < .05, effect size = 0.65). Prediction accuracy of Random Forest (RF) and eXtreme Gradient Boosting (XGBoost) for priority classes were 0.912 and 0.954, respectively. Both models exhibited lake surface area as the variable with the highest importance. CPI generally increased with a decrease in lake surface area. This was attributed to less ecological substitutability and higher exposure levels of anthropogenic stressors such as pollution to a species in smaller lakes. Also, the highest species richness per unit area was recorded for high‐priority lakes. Thus, smaller habitats or lakes may be prioritized for conservation although larger waterbodies or habitats should not be ignored. The index can be customized to local, regional, and international scales as well as marine and terrestrial habitats.

## Graphical abstract

We developed and tested a novel conservation priority index (CPI) that ranks habitats to aid in prioritizing them for conservation. The species richness, endemicity, threat status, and surface area were incorporated in the index.

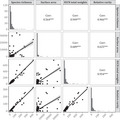

## 1 INTRODUCTION

Human‐induced multiple stressors are exacerbating global biodiversity loss (Dobson, 1992; Dudgeon et al., 2006; Reid et al., 2019) and persistently altering ecosystem to function and provide services such as flood mitigation and food (Hooper et al., 2005; O’Connor & Crowe, 2005). These stressors, including climate change, habitat degradation, pollution, species invasions, and overexploitation, exhibit synergistic impacts on the ecosystems (Brook, 2008; Craig et al., 2017; Dudgeon et al., 2006; Hermoso et al., 2009), thereby increasing the complexity in managing and monitoring the ecosystem integrity (Craig et al., 2017). New communities are created within ecosystems (Pandolfi et al., 2020); loss of species spatial insurance is observed (Thompson et al., 2017); and ecological interactions within ecosystem are impeded, affecting species survival and ecosystem functioning and consequently increasing biodiversity loss (De Bernardi, 1981).

Freshwater biodiversity loss has outpaced both terrestrial and marine ecosystems (Collen et al., 2014; Dudgeon et al., 2006; WWF, 2020). Contrarily, efforts to reduce defaunation are mostly directed towards the two latter ecosystems (Abell, 2002; Tickner et al., 2020), which are poor surrogates for conserving freshwater biodiversity (Darwall et al., 2011). Furthermore, due to high levels of endemism, freshwater species have less ecological substitutability when habitats are lost, fragmented, polluted, or invaded by exotic species (Abell, 2002; Arthington et al., 2016; Dudgeon et al., 2006). In addition, because freshwater ecosystems provide myriad ecosystem services to humans (McIntyre et al., 2016), they are predisposed to drastic and intermittent reclamation, increased pollution, and overexploitation (Hermoso et al., 2009). As a result, freshwater species have reduced by 76% in the last 50 years compared with 39% decline for marine and terrestrial populations (WWF, 2020). Of the 29,500 freshwater species assessed by the International Union for Conservation of Nature (IUCN), 27% are threatened with extinction and megafauna populations have declined by 88% from 1970 to 2012 (Tickner et al., 2020).

Freshwater biodiversity has consistently continued to decline (Hermoso et al., 2016), despite the proliferation in development and use of different conservation management strategies such as conservation planning tools, priority indices, and protected areas (Hermoso et al., 2016). The anomaly is alluded to the inability to manage freshwater protected areas, political interference, poor sensitization, and poor delineation (Bastin et al., 2019; Hermoso et al., 2016; Holland et al., 2012). Also, the ecological integrity is mostly assessed at species level rather than the whole ecosystem (Vié et al., 2008), albeit habitat loss and degradation being the major catalyst to freshwater biodiversity loss (Dudgeon et al., 2006; Vié et al., 2008). Furthermore, conservation priorities are mostly inclined to large waterbodies because of their high species richness, endemism, and threatened species (Sayer et al., 2018). Likewise, most studies are skewed to large waterbodies (Biggs et al., 2017), despite the significance of small waterbodies as refugia for threatened species (Biggs et al., 2017; Olwa et al., 2020). Besides species richness, ecosystem parameters such as surface area need to be incorporated in designing freshwater conservation strategies (Grzybowski & Glińska‐Lewczuk, 2019). Also, ecosystem competing interests and costs should be considered (Dudgeon et al., 2006; Nieto et al., 2017). For effective conservation, the catchment may need to be included when delineating areas for conservation (Dudgeon et al., 2006; Nieto et al., 2017), except that there is high cost associated with conserving even small catchment (Dudgeon et al., 2006).

In this context, priority‐based approaches are necessary to rank different habitats or waterbodies at high risk of degradation, but also practically viable for conservation (Howard et al., 2018). Elsewhere, prioritization indices have been implemented for caves in Brazilian Atlantic Rain Forest (Souza Silva et al., 2014); forests between Atlantic forest and Cerrado (de Mello et al., 2016), and rivers in the Mediterranean basin (Hermoso et al., 2009) to ensure preferential ecosystem selection for conservation. Freshwater ecosystems have not been widely considered as most of the studies have been waterbody or habitat specific, with limited information to rank them for site‐based conservation. The paucity of data on most taxa, including fishes, had in the past curtailed broader‐scale distribution analysis. However, recently, substantial amounts of data on the occurrences of different taxa have been made freely available in online repositories such as the Global Biodiversity Information Facility portal and International Union for Conservation of Nature (IUCN). This study aims to construct and test a novel conservation priority index on the fish species distribution from African lakes. We apply two model ensemble methods, eXtreme gradient boosting (XGBoost) and Random Forest (RF), to determine the most important variables in ranking the lakes for conservation. The index will aid in preferentially selecting ecosystems for site‐based conservation, especially where resources are limiting.

## 2 METHODS

### 2.1 Study area, data acquisition, and processing

**FIGURE 1:**
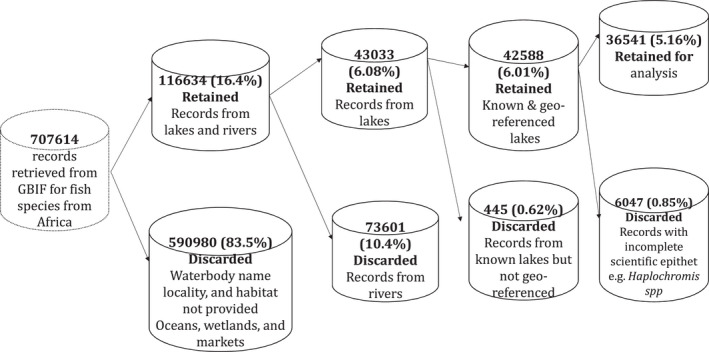
Data filtering pipeline for records obtained from GBIF (2020). GBIF, Global Biodiversity Information Facility

We considered fish species records from all the lakes in Africa found in the Global Biodiversity Information Facility (GBIF, 2020). We retrieved the records with the *occ_download_get* function in *rgbif* package (Chamberlain et al., 2020), and genera names were changed in conformity with FishBase nomenclature (Froese & Pauly, 2021), which is based on Van Oijen (1996). We used the coordinates to correctly reference the records that were outside the geographic range described in FishBase. For instance, *Haplochromis eduardii* records that were found in Lake Albert in GBIF data were moved to Lake Edward, where the species is endemic (Froese & Pauly, 2021). Records with incomplete scientific epithets such as *Oreochromis* spp. and *Thoracochromis* spp. were discarded. Records without a lake or waterbody of origin, but with coordinates, were geo‐referenced using Google Earth Pro or used habitat descriptions, verbatim locality, and location remarks (Figure 1).

To avoid duplication of lakes, we changed all lake nomenclature to their English names; for example, changing “lac Edouard” to Lake Edward. However, presently accepted names were maintained for lakes whose names have changed over time.

To retrieve the species IUCN conservation status from IUCN Red List database (www.iucnredlist.org), we used *iucn_summary* and *iucn_status* functions in the *taxize* R package (Chamberlain et al., 2020). The species were classified according to IUCN Red List for threatened species (IUCN, 2012). Species with IUCN status of LR/nt were changed to near threatened (NT).

**FIGURE 2:**
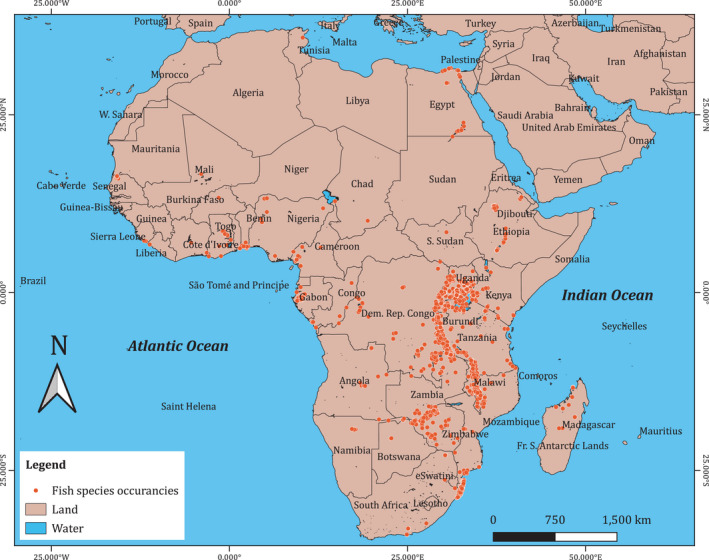
Map showing the georeferenced fish species 42,588 records from 273 inland Africa lakes (Data obtained from GBIF, 2020)

**FIGURE 3:**
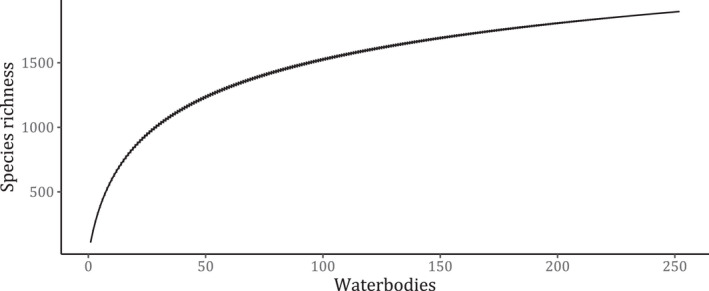
Species accumulation curve for the species obtained from the lakes in Africa

The data retrieved were processed through a pipeline and only 36,541 (5.16%) records were retained for analysis (Figure 1). We used a species accumulation curve to evaluate whether most of the fish species from African lakes were represented in our data in order to test the conservation priority index (Figure 3). Fish species richness was determined for each lake. Both waterbody relative rarity and total IUCN weighting were computed for each species found in a particular lake and country. The distribution of analyzed fish species records was mapped (Figure 2).

### 2.2 Evaluating species relative rarity among waterbodies or habitats

Conservation strategies, laws, policies, and binding targets are mostly drawn at regional and international conventions such as Convention of Biological Diversity (CBD), and Ramsar Convention, Convention on International Trade in Endangered Species of Wild Fauna and Flora. However, national authorities or parties are vital in implementing or enforcing the treaties, laws, and policies such as CBD (1992). Thus, the species relative rarity, and consequently the conservation priority index, were computed at a national level.

We computed the species relative rarity (SRR) of each species as the ratio of the number of waterbodies where the species was found in a particular country to total number of waterbodies with fish species data and correctly georeferenced in that country (Equation 1). We introduced an arbitrary value of 1 to convert the ratio to be highest for the rarest species and approach 0 if a species is found in most of the waterbodies (Equation 1). We assumed that if a species is found in many waterbodies, it has a greater area of occupancy, and thus not threatened by a single stressor. In IUCN, such a species is listed as least concern (IUCN, 2012). Furthermore, a species that is threatened but found in many waterbodies or habitats would have a low relative rarity compared with a least concern species found in only one lake or waterbody or habitat. The relative rarity weight for a waterbody was computed as the sum of rarity weights for the species in that waterbody scaled to the total number of species in the same waterbody (Equation 1). Species relative rarity (SRR) for a species was computed as:

(1) $$\text{SSR}=1‐\frac{\mathrm{Ws}}{\mathrm{Wt}}$$

*Ws* is the total number of lakes where the species was observed in particular country; and *Wt* is the total number of correctly referenced lakes obtained in particular country. The conservation priority index for the waterbody will be 0 if only one waterbody is considered in a particular country. Relative rarity for each waterbody is the summation of the relative rarity for each species found in that waterbody. Relative rarity for waterbody was computed as follows:

(2) $$\text{RRw}=\sum_{n=1}^{i}\text{SSR}$$

### 2.3 Incorporating waterbody or habitat surface area in the conservation priority index

Implementation of conservation strategies is often constrained by financial resources. Also, most countries invest less in biodiversity conservation compared with other sectors such as agriculture, industrialization, and infrastructure development (Bayon et al., 2000). Thus, before delineating any lake or habitat for conservation, its surface area should be considered to ensure unit costs per unit area are known. In developing this index, the unit cost could not be established, thus surface area was incorporated as a proxy of the financial implications if the waterbody or habitat is prioritized for conservation. Indirectly, the competing interests such as food provision are implied because most of the commercial fishing activities such as trawling are conducted in larger waterbodies. Rivers were not included in the index because the hydro‐geomorphological transformations such as damming create distinct ecosystems along the river course that would require to conserve a particular section independently.

In the study, for each correctly referenced waterbody, we collated its surface area from literature (Burgis & Symoens, 1987; Ogutu‐Ohwayo et al., 1999; Olowo et al., 2004; Schofield & Chapman, 1999; Vanden Bossche & Bernacsek, 1990). Where it was not possible to get this information from literature, we used the coordinates to locate the lake on Google Earth and approximated its surface area. This method was applied to lakes Gawa, Kabaleka, Wamala, Nakabale, Owapet, Kirimira, Gashana, Birengero, Igombe, Blue Lake, Carumbo, Chahafi, Rwanyakizinga, Mirayi, Maxai, Sheba Kelbia, Avanga, Chidya, Nkuruba, and Kabaka. The surface area of lakes Natuali, Chankaranga, Okurachere, Kasunju, and Mutabyo could not be determined from both literature and Google Earth, and these lakes were discarded. These are mostly minor lakes that have not been georeferenced on Google Earth. We cross‐referenced all records in lakes with the country of origin; for example, where records from Lake Victoria had country of origin different from Uganda, Kenya, or Tanzania were discarded or georeferenced using Google Earth to determine the country or lake of origin. For shared lakes, such as Tanganyika, Victoria, Chala, Nyasa, Cyohoha, Albert, Kivu, Mweru, Rweru, Jipe, Edward, Turkana, and Kariba, the surface area was approximated for the portion shared in each country. We considered the country‐specific surface area because the conservation funding would possibly be determined by the area of national jurisdiction (CBD, 1992).

### 2.4 Incorporating species' IUCN Red List status in the conservation priority index

The IUCN Red List for threatened species is the most comprehensive and detailed databases that have evaluated the species’ threat levels worldwide (Vié et al., 2008). The species evaluations are conducted at national, regional. or international levels to inform policies, laws, and targets (Vié et al., 2008). Most indices or conservation strategies are designed to cater for threatened species, while excluding data deficient, least concern, near threatened, and not evaluated species. This anomaly predisposes mostly not evaluated (NE) and data deficient (DD) species to become extinct unknowingly. Thus, IUCN suggested that DD and NE species should be considered as critically endangered until their status is known (IUCN, 2012). For least concern species, managers have put less effort to monitor the trends of their stock sizes. For example, species such as *Labeo victorianus* and *Oreochromis esculentus* which once dominated the commercial stocks in Lake Victoria (Cadwalladr, 1965) are now critically endangered (FishBase team RMCA & Geelhand, 2016). Thus, in this index, all species were considered and weights were given based on the threat level: highest and lowest weights assigned to extinct and least concern conservation categories, respectively. The weights were assigned as follows: ET = 7, EXw = 6, CR = 5, DD = 5, NE = 5, EN = 4, VU = 3, NT = 2, and LC = 1. We computed a conservation score (Cwt) as the product of total number of species in a given IUCN Red List category and weight assigned to that threat category, summed across all the IUCN Red List categories (Equation 3).

(3) $$\text{Cwt}=\sum_{1=1}^{n}IUCN_{\mathrm{Rt}}*IUCN_{w}$$

The conservation priority index (CPI) was then formulated as a product of the conservation score (IUCN total weights) and relative rarity for each species, summed across all of the species within a lake, divided by the area of the lake, and a scaling constant to account for the number of categories (Equation 4). Here, we used lake area as a penalty based on several assumptions. (1) The cost of conservation is generally higher for large lakes compared to small lakes. (2) Biodiversity generally have limited room for adaptation to stressors in small waterbodies than large waterbodies. (3) Conservation actions are likely to be more effective in small lakes than in large waterbodies. These assumptions aimed at controlling for the size of the waterbody so that the index is not necessarily higher for larger lakes.

(4) $$CPI_{w}=\frac{Cwt*RRw}{A_{W}*8}$$

Where, for each waterbody: Aw is the total surface area of the waterbody. Cwt and RRw are the IUCN total weights and relative rarity for a particular waterbody. The value of 8 was a scaling constant indicating the total number of IUCN categories considered. Thus, the scaling constant depends on the IUCN categories the user considers in the index.

### 2.5 Significance of the variables on the performance of the index

We used Pearson correlation to determine the relationship among species richness, IUCN total weighting, surface area, and waterbody relative rarity. To determine the relative importance of the variables in the conservation priority index (CPI), we arbitrarily classified the waterbody as high priority (CP1 > 0.5, *n* = 56) and low priority (CPI < 0.5, *n* = 217). We compared two ensemble machine learning algorithms: Random Forest (RF) and eXtreme Gradient Boosting (XGBoost) to develop model classification predictions for the priority classes (high and low). We converted the categorical variable (country where the lakes were found) into dummy variables (numerical codes) using One‐Hot Encoding, and this variable was included in the model to account for national variations in species composition and conservation status. The pre‐processed data were randomly partitioned into training (70%) and testing (30%). Random Forest (RF) training model was tuned with *tuneRF* function in *randomForest* package with a step factor of 2, and an improve rate of 0.05 (Liaw & Wiener, 2002). Partial dependence plots were used to indicate how the index varied with changes in the index parameters, namely conservation score (IUCN total weights, waterbody relative rarity, country, and surface area). For XGBoost, parameters such as mglogloss (evaluation metric) and softprob (objective) were included in the watch list prior to constructing the best model. The model performance was measured using metrics including recall (sensitivity), specificity, precision, F1‐score, and accuracy (Yokoyama & Yamaguchi, 2020).

For both models, variable importance plot was determined. We used both XGBoost (tree combinations at the start) and RF (independent tree building) to compare the predictability accuracy and identify the best algorithms to classify the data. Both algorithms are suitable for variances on the input data, and can handle overfitting with variation on the hyperparameter tuning. We used the Wilcoxon signed‐rank test to compare the differences in the median surface area, IUCN total weights, and the waterbody relative rarity for the two priority classes (high and low). The effect size was computed with the *wilcox_effsize* function from *rstatix* package.

## 3 RESULTS

### 3.1 Species composition, richness, and relationship among index variables

A total of 1897 species were recorded from 273 lakes in 34 countries of Africa. Uganda had the highest number of lakes assessed (39), Democratic Republic of Congo (26), and Rwanda (25). The species accumulation curve increased at a low rate after 20 lakes (Figure 3). Lake Nyasa had the highest species richness (424), followed by lakes Tanganyika (391), Nokoué (246), Victoria (216), and Ahémé (216). In lakes Gashanga, Kingiri, and Saka, only one fish species was observed in the assessed GBIF data. Of 1897 species, 1269 (66.9%) were least concern, 209 (11.0%) not evaluated, 160 (8.4%) data deficient, 81 (4.1%) vulnerable, 92 (4.8%) critically endangered, 45 (2.4%) endangered, 39 (2.1%) near threatened, and 2 (0.1%) were extinct.

**FIGURE 4:**
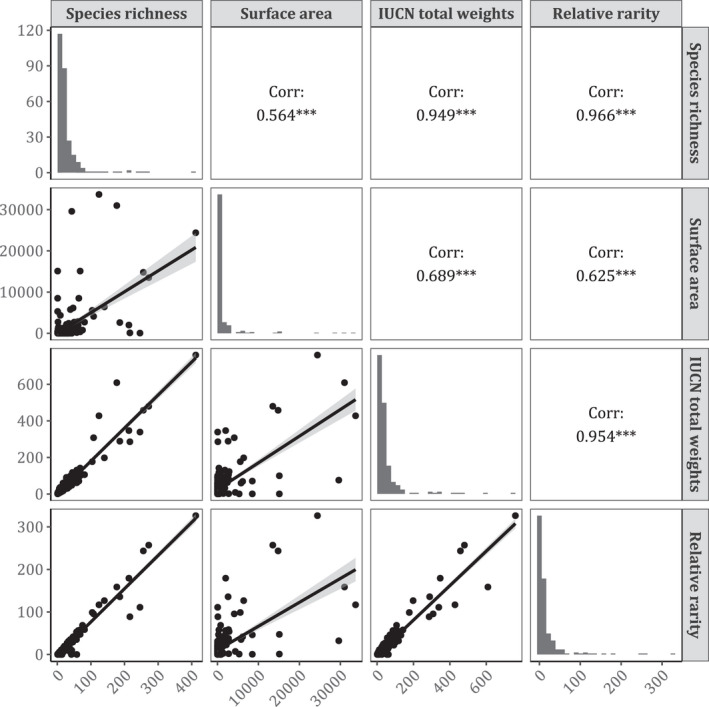
Correlation coefficient of variables incorporated in the conservation priority index (***p value < .001)

Malawi side of Lake Nyasa had the highest IUCN weighting of 760 followed by Lake Victoria‐ Uganda (609); Lake Tanganyika‐Tanzania (480); and Lake Tanganyika‐DRC (458). Lake Nyasa‐ Malawi, had the highest relative rarity (362.5) followed by Lake Tanganyika‐Tanzania (257.2); Lake Tanganyika‐DRC (243.3); and Lake Tanganyika‐Zambia (179) (Appendix 1). In contrast, Lakes Otjikoto and Guinas had the highest species richness per unit surface area of 800 and 303 individuals/km^2^, respectively (Table 1). Strong positive correlation was observed between IUCN total weights and species richness (*r* = .944, *df* = 271, *p* < .001), and relative rarity and species richness (*r* = .966, *df* = 271, *p* < .001). However, moderate relationship was observed between surface area and richness (*r* = .564, *df* = 271, *p* < .001), and IUCN total weights (*r* = .689, *df* = 271, *p* < .001) and relative rarity (*r* = .625, *df* = 271, *p* < .001) (Figure 4).

**TABLE 1:** Model classification performance for index priority classes in testing data (*n* = 91)

Model	Accuracy	Recall/Sensitivity	Specificity	Precision	F1 Score
RF	0.912	0.933	0.813	0.958	0.954
XGBoost	0.9341	0.947	0.875	0.972	0.959

Abbreviations: RF, Random Forest; XGBoost, eXtreme Gradient Boosting.

### 3.2 Conservation priority index, classification of lakes, and model predictions

Lakes Otjikoto and Guinas from Namibia had the highest conservation priority index of 137.5 and 52.1, respectively, followed by Lake Nkuruba in Uganda at 34.1 (Appendix 1). CPI for lakes Ngami, Piso, and Faguibine was zero (Appendix 1). After classifying the lake into 2 priority conservation classes, of 273 lakes, 56 (20.5%) were of high conservation priority class and 217 (79.5%) with low priority (Appendix 1). Uganda had the highest number of lakes with high priority (14), followed by Cameroon, Madagascar, and South Africa (6) (Appendix 1). The median surface area (km^2^) for low priority class (64) was significantly higher than that of high priority class (1) (Wilcoxon signed rank: W = 11,768, *p* < .05, *n*1 = 217, *n*2 = 56, effect size = 0.65). The median IUCN total weights and waterbody relative rarity for both low and high priority classes were not significantly different (IUCN: W = 5840, *p* = .66 and RRw: W = 5522, *p* = .29).

**FIGURE 5:**
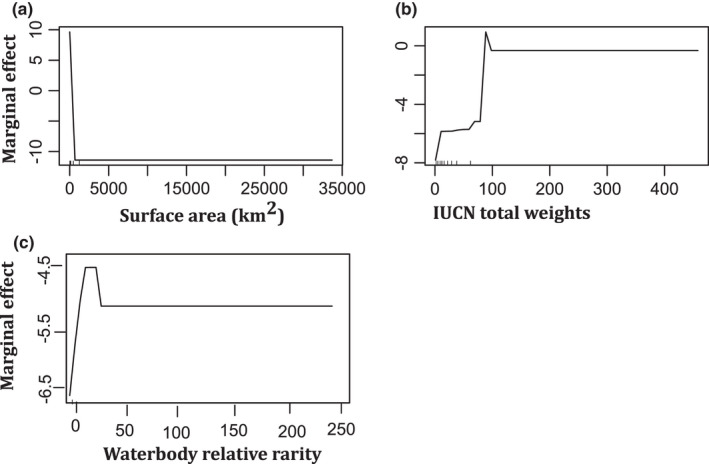
Partial dependence plots for testing data of the variables on predicting high priority classifications (a: Lake surface area (km^2^), b: IUCN total weights; c: waterbody relative rarity)

XGBoost model had the highest model classification performance with an accuracy of 0.934 (95% CI: 0.86, 0.98) for test data compared with 0.921 (95% CI: (0.834, 0.96) for Random Forest (RF) (Table 1). However, both models had similar F1 score of 0.959 for XGBoost and 0.954 for Random Forest (RF). The partial dependence plots with test data using RF for priority classes varied depending on the variables (Figure 5). When the surface area of the lake was small, the model predicted a high priority class (Figure 5a). In contrast to surface area, the increase in both IUCN total weights and waterbody relative rarity led to prediction of high priority class for the lakes by RF model (Figure 5b,c). The model prediction for priority classes versus the 34 country codes was not presented in plots.

**FIGURE 6:**
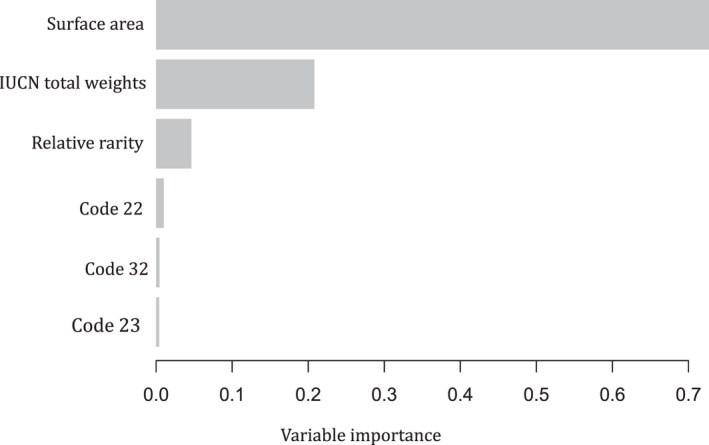
Variable importance: Gain for XGBoost (code22: Uganda; code32: Rwanda; and code23: Nigeria)

**FIGURE 7:**
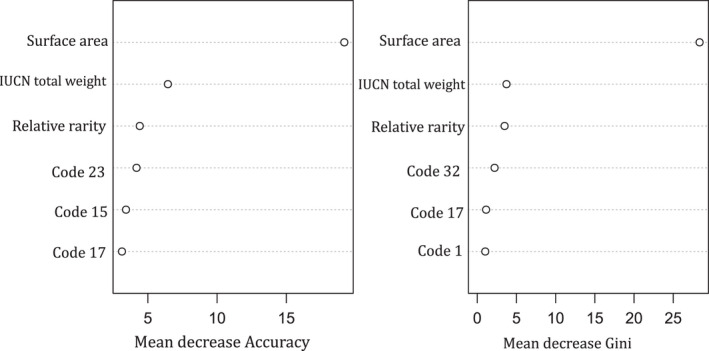
Variable importance (mean decrease in accuracy and mean decrease in Gini) for the Random Forest model (Country codes: code 23: Uganda; Code 15: Kenya; Code 17: Madagascar; Code 32: Rwanda; and Code 1: Angola)

In both models, surface area had the highest variable importance (19.2% mean decrease in accuracy and 29.3% mean decrease in Gini for RF) and 72.7% Gain for XGBoost (Figures 6 and 7). In RF, surface area was followed by IUCN total weights (6.44%), rarity (4.4%), and least for country codes. Similarly, in XGBoost, surface area was followed by IUCN total weights (20.8%), waterbody relative rarity, and country codes. In both models, the country variable was converted into dummy codes and only three country codes were significant and displayed in the plot (Figures 6 and 7).

## 4 DISCUSSION

### 4.1 Species occurrences, rarity, and relationship among index variables

A total of 1897 fish species from 36,541 occurrence records were included in this index, representing 64% of all known freshwater fish species in Africa (Eschmeyer, 2005). The species accumulation curve approached asymptote, which suggested that most of the species were considered in the experimentation of the index (Gotelli & Colwell, 2001). Uganda followed by DRC had the highest number of lakes considered in the index, which could be attributed to extensive open source data sharing in the GBIF compared to other countries (PBES Technical Support Unit on Knowledge & Data, 2020). Some data points shared in the portal were filtered out in the index because key attributes such as waterbody name, geolocation, site description, habitat description, and verbatim locality were not provided, which reduced the number of occurrences considered in the index.

Lakes Nyasa, Victoria, Tanganyika, Nokoué, and Ahémé had the highest species richness, while lakes including Kingiri, Saka, and Gashanga had the least. However, lakes Giunas and Otijkoto had the highest species richness per unit surface area. Also, strong positive correlations were observed among the index variables. The strong relationship between species richness and lake surface area agreed to the biogeographical principles that the larger the habitat, the more species it harbors (Rosenzweig, 1995). Differences in species richness and relative rarity among waterbodies are attributed to geomorphological, abiotic, and biotic factors (Brown et al., 2007). Also, isolated inland waterbodies favor rapid allopatric speciation and adaptive radiation because the species are exposed to different evolutionary pressures (Basiita et al., 2018). Lakes Nyasa, Victoria, and Tanganyika are endowed with diversity haplochromine cichlids, which has been attributed to the rapid speciation rates associated with habitat heterogeneity and disruptive sexual selection (Salzburger et al., 2005; Seehausen, 2000). The haplochromine lineages are endemic to Lake Tanganyika (Salzburger et al., 2005). The geomorphological barriers, for example, the Murchison Falls along Victoria Nile hindered fish migration to Lake Kyoga from Albert (Basiita et al., 2018). The falls along River Semiliki prevent fish passage to Lake Albert from Edward (Acere & Mwene‐Beyanga, 1990). Similarly, Lake Victoria and Kyoga were previously separated by the Owen and Bujagali falls (Basiita et al., 2018), and a sandbar separated Lake Nabugabo and Victoria (Stager et al., 2005). These biogeographical barriers may have led to allopatric speciation. For example, Lake Nabugabo which was once connected to Lake Victoria had five endemic species (van Alphen et al., 2004; Ogutu‐Ohwayo, 1993).

The high species richness of Lake Nokoué could be attributed to its high habitat and seasonal variability; for example, the estuarine, freshwater, and marine habits (Lalèyè et al., 2003). The lake is connected to Atlantic Ocean, Porto‐Novo lagoon (30 km^2^), and the Ouémé Delta (Lalèyè et al., 2003). These habitats support a diversity of species within the lake. However, Lalèyè et al. (2003) identified only 51 species from Lake Nokoué while understanding its spatial and seasonal ichthyofaunal distribution. GBIF holds data from different sources, and thus the 216 species obtained from the data could be attributed to different data sources. Lake Nokoué, similar to lakes Nyasa, Victoria, and Tanganyika is threatened by anthropogenic pressure due to intense settlement around them. According to the Global Nature Fund (2019), along the Cotonou channel to the Atlantic Ocean was surrounded by about 700,000 people who depended on the lake for food and water.

In the study, 66.9% of the species were classified as least concern under the global IUCN Red List for threatened species, which accounts for the low conservation priority indices. Although most of the species were not endangered, localized threats to the individual species cannot be ignored. National Red Lists should be developed to ensure effective monitoring of the species threat status. For example, species such as *Mormyrus kanuume* is exploited around Lake Victoria as bait in the *Lates niloticus* fishery (Bassa, 2018), which would threaten its population at national and regional levels. In Uganda, a National Red List was produced for all other taxa except fish (MTWA, 2018). Differences were observed in the species threat levels between the global and national lists; for example, *Oreotragus oreotragus* (Klipspringer), *Otomys typus* (Northern Groove toothed Rat), *Scotophilus leucogaster* (Northern Lesser House Bat and White‐bellied Yellow Bat) were regarded as least concern by global IUCN Red List but evaluated as vulnerable in Uganda National Red List (MTWA, 2018). In South Africa, *Kniphofia leucocephala* (Red‐hot Pokers) are not evaluated under the global IUCN Red List but critically endangered in South Africa (SANBI, 2010).

### 4.2 Conservation priority index and model predictions

Lakes Giunas and Otjikoto had the highest conservation priority index. The lakes harbor *Tilapia guinasana*—a critically endangered species introduced from Lakes Giunas to Otjikoto (Skelton, 1978). Generally, the restricted size of the lakes increases the vulnerability of their biota to sudden changes in ecological pressures such as alien species invasion and habitat degradation (Irish, 1991; Skelton, 1978). For example, *T. guinasana* was introduced to Lake Otjikoto from Giunas and invaded the niches for populations of *Pseudocrenilabrus philander* (Irish, 1991). Both lakes are threatened by habitat degradation and pollution because they are surrounded by agricultural fields (Irish, 1991). Lake Otjikoto was declared a national monument in Namibia, and the only underwater museum in the world because ammunitions were dumped in the lake after World War 1 (van Rooyen, 2010). In addition, both lakes are the only permanent lakes in Namibia (ATFALCO, 2012) and thus requires urgent conservation measures both nationally and globally. Noticeably, the Ramsar Secretariat (2002) noted that the two sinkhole lakes exclusively qualify because of the composition of threatened fish species.

Although lakes Nyasa, Victoria, Tanganyika, and Nokoué had the highest species richness, their CPI were low. The index considered surface area as a proxy for the cost of implementing a biodiversity strategy that would be applied on the lake. According to article 5a of the Convention on Biological Diversity multilateral treaty, each contracting party shall “develop national strategies, plans or programmes for the conservation and sustainable use of biological diversity or adapt for this purpose existing strategies plans or programmes which shall reflect, inter alia, the measures set out in this Convention relevant to the Contracting Party concerned.” Therefore, the cost involved in conserving Lake Tanganyika, which is shared by four countries, would require harmonized national strategies. Also, because the surface area is large, ecological variabilities are available for the species to seek refugia if its native habitat or niche is affected or invaded by a predator. For example, in Lake Victoria, rocky dwelling *Paralabidochromis* species were not highly affected by the invasion of *Lates niloticus* (Balirwa et al., 2003). In small lakes or habitats, due to less ecological variability, the species are highly exposed to ecological threats. Also, it requires less costs to conserve small habitats and local community conservation approach can easily be applied. The CPI of the lake was zero if it was only one lake or habitat assessed in a particular country, thus no comparisons could be made for priority selection.

### 4.3 Classification and prediction of habitat priority

Random Forest (RF) and eXtreme Gradient Boosting (XGBoost) had similar model prediction accuracy, sensitivity (recall), F1 score, precision, and specificity for both training and testing data. After classification of the lakes into high and low priority, the partial dependence plots from RF models showed that high priority classes were predicted at smaller surface area of the lake. The higher the surface, the lower the priority for conservation. Partial dependence plots are vital in determining the relationship between the variables and the predicted probabilities of the classes (Cutler et al., 2007).

Surface area was ranked as the variable with highest importance in the index by both models. Although the species rarity, richness, and threat status are vital biodiversity conservation science, the ability and success of managing the habitat will depend on the costs required to implement the strategies. Biodiversity funding mostly in developing countries and world over is still minimal (Bayon et al., 2000), despite the 33 trillion US dollars that is averagely generated from ecosystem services annually (Costanza et al., 1997). The IUCN key biodiversity areas (KBA) are sites vital to protect global biodiversity dependent on key trigger species (IUCN, 2016). However, priority areas for conservation such as protected area can lie beyond key biodiversity areas (IUCN, 2016). Similar to study, IUCN (2016) noted that for conservation priority areas, the cost, connectivity, and evolutionary history should be considered.

## 5 CONCLUSION

The aim of this study was to construct a novel conservation priority index to aid in selection of a habitat for site‐based conservation. The index was designed and tested on 1897 fish species from 273 Africa inland lakes in 34 countries. We applied two model ensemble methods, eXtreme gradient boosting (XGBoost) and Random Forest (RF), to determine the most important variables in ranking the lakes for conservation. Results showed that lake surface area was the most important variable for ranking habitats for site‐based conservation. While species richness is generally higher for large lakes compared to small ones, this study suggests that smaller waterbodies need to be prioritized for because of the low habitat heterogeneity, low ecological substitutability for the species, and higher levels of exposure to human‐induced threats in small waterbodies compared to large systems. For large systems with vast habitat heterogeneity, fish species can easily seek refugia in other habitats. This index can be applied at local, national, and regional scale for other taxa, and can aid in preferentially selecting ecosystems for site‐based conservation, especially where resources are limiting. This index can be greatly affected by incorrect identification of the species. Also, species need to be correctly geolocated to avoid over‐ or underweighting/ranking of the waterbody, habitat, or any ecosystem.

## CONFLICT OF INTEREST

The authors declare no conflict of interest.

## AUTHOR CONTRIBUTIONS

**Anthony Basooma:** Conceptualization (lead); Data curation (lead); Formal analysis (lead); Investigation (lead); Visualization (lead); Writing – original draft (lead); Writing – review & editing (lead). **Herbert Nakiyende:** Conceptualization (equal); Methodology (equal); Writing – review & editing (equal). **Mark Olokotum:** Conceptualization (equal); Investigation (equal); Methodology (equal); Validation (equal); Writing – review & editing (equal). **John S. Balirwa:** Conceptualization (equal); Methodology (equal); Writing – review & editing (equal). **Winnie Nkalubo:** Investigation (equal); Methodology (equal); Writing – review & editing (supporting). **Laban Musinguzi:** Conceptualization (equal); Investigation (equal); Methodology (equal); Validation (equal); Writing – review & editing (equal). **Vianny Natugonza:** Conceptualization (equal); Investigation (equal); Methodology (equal); Supervision (lead); Validation (equal); Writing – review & editing (equal).

### OPEN RESEARCH BADGES

This article has earned an Open Data Badge for making publicly available the digitally‐shareable data necessary to reproduce the reported results. The data is available at [https://doi.org/10.5061/dryad.4b8gthtcx].

## Data Availability

The data can be accessed on Dryad (DOI https://doi.org/10.5061/dryad.4b8gthtcx).

## APPENDIX 1

### Species richness, surface area, IUCN total weights, relative rarity, and priority index

**Table:**

Country	Lake	Species richness	Surface area (km^2^)	Richness/SA (ind/km^2^)	IUCN total weights	Relative rarity	CPI
Angola	Cuanavale	30	0.95	32	54	20	5
	Salia Kuembo	14	0.56	25	18	7	1.9
	Cuito Source	6	0.49	12	10	2.3	0.9
	Calundo	28	4.2	7	38	18.3	0.8
	Carumbo	6	2.1	3	18	3.5	0.7
Benin	Hlan	43	1.9	23	48	9.8	0.9
Cameroon	Ejagham	8	0.49	16	36	7.2	8.3
	Bemin	6	0.58	10	30	5.4	5.8
	Barombi Koto	27	3.3	8	91	22.3	2.8
	Barombi	17	5	3	60	13.4	1.2
	Soden	5	1.33	4	9	4	0.6
	Manengouba	2	0.37	5	2	1.8	0.6
Congo	Blue	15	0.12	125	19	11.8	15.8
	Yangala	10	1.82	5	12	7.3	0.6
DRC	Lukushi	23	2.66	9	28	19.5	1.1
	Fwa	12	2.13	6	12	11	0.6
Gabon	Kayaus	13	1.14	11	21	4.9	0.8
	Nguene	25	3.76	7	41	10.9	0.6
Madagascar	Sarodrano	3	0.1	30	7	2.2	7.2
	Ravelobe	9	0.36	25	21	6.4	5.1
	Djabala	2	0.1	20	7	0.9	4.2
	Andjavibe	3	0.31	10	8	1.8	1.7
	Amparihibe	4	1.38	3	11	2.3	0.6
	Andrapongy	10	4.69	2	29	6.9	0.6
Malawi	Chikukutu	22	2.5	9	26	9.7	0.6
Mozambique	Maxai	6	0.41	15	16	4.8	3.9
Namibia	Otjikoto	4	0.005	800	10	2	137.5
	Guinas	2	0.0066	303	6	0.8	52.1
Nigeria	Isemu	35	0.12	292	39	21.5	25.5
	Kware	17	0.17	100	17	8.8	6.4
Rwanda	Kisantu	8	0.93	9	8	5.9	0.8
Sierra Leone	Kwako	20	1	20	25	9.5	1.5
South Africa	Shazibe	8	0.1	80	15	4.1	9.2
	Makhawulani	24	1	24	31	20.2	3.2
	Kuzilonde	11	0.83	13	23	6.9	2.5
	Bhangazi	19	1	19	30	11.8	2.1
	Mgobezeleni	20	1.28	16	35	12.8	2.1
	Shengeza	9	1.22	7	19	3.9	0.9
Tanzania	Nala	3	0.22	14	7	2.4	3.5
	Malimbe	4	0.27	15	8	2.9	2.4
	Chala‐TZ	4	2.6	2	16	3.2	0.7
Uganda	Nkuruba	3	0.02	150	7	1.8	34.1
	Kayugi	12	0.25	48	23	7.4	7.4
	Manywa	8	0.25	32	17	4.5	5.1
	Gigate	20	1.7	12	58	12.3	2.9
	Kayanja	17	1.2	14	33	10.5	2.3
	Naragaga	13	1.98	7	33	8.2	1.4
	Agu	24	4	6	62	15.6	1.4
	Nawampasa	32	8	4	92	21.9	1.1
	Kawi	24	5	5	58	15.6	1
	Kabaka's	1	0.1	10	1	0.8	1
	Kasodo	4	0.71	6	8	2.3	0.9
	Gawa	3	1.42	2	11	2.2	0.7
	Kabaleka	5	1.14	4	7	3.3	0.6
	Kimira	8	2.21	4	16	4.1	0.5
Zambia	Chunga	4	0.19	21	4	1.6	1.1
Angola	Dilolo	3	18.9	0.159	3	1	0.007
Benin	Nokoué	246	49	5.02	339	111	0.41
	Lake Ahémé	216	100	2.16	286	88.8	0.152
	Toho	37	9.52	3.887	46	7	0.121
Botswana	Ngami	42	154	0.273	54	0	0
Burkina Faso	Bam	7	20	0.35	7	0	0
Burundi	Rweru‐BI	15	80	0.188	26	5.8	0.018
	Tanganyika‐BI	186	2600	0.072	289	135.8	0.01
	Cyohoha‐BI	9	55	0.164	13	1.3	0.004
	Rugwero	10	100	0.1	15	1.8	0.003
Cameroon	Douloumi	7	7.79	0.899	7	6.3	0.101
	Nyos	1	1.58	0.633	1	0.9	0.071
	Ossa	6	39.27	0.153	10	4.9	0.026
	Mbakaou	3	500	0.006	7	2.6	0.001
Chad	Iro	5	110	0.045	5	2.5	0.003
	Chad	7	1350	0.005	11	3.5	0.001
Congo	Kobambi	12	3.24	3.704	12	8.3	0.322
	Telle	15	20	0.75	27	12.2	0.139
	Youbi	4	3.5	1.143	4	3	0.107
	Cayo	4	18.9	0.212	8	3	0.042
Côte d'Ivoire	Kossou	30	178	0.169	31	11.5	0.008
	Ayame	14	110	0.127	15	3.5	0.005
DRC	Ndaraga	3	3.73	0.804	7	2.8	0.222

Mukamba	3	6.7	0.448	3	2.3	0.044

Lac Nkolentulu	1	25.3	0.04	5	1	0.024

Tumba	75	765	0.098	107	68.6	0.016

Mulenda	8	62	0.129	8	6.6	0.013

Yandja	19	217	0.088	25	17.1	0.013

Kisale	29	300	0.097	34	24.2	0.012

Upemba	45	530	0.085	56	39.1	0.012

Kabamba	13	126	0.103	14	10.8	0.012

Zimbambo	5	189	0.026	10	4.5	0.006

Edward‐DRC	33	1650	0.02	80	30.9	0.006

Mai‐Ndombe	57	2305	0.025	101	52.8	0.005

Mweru‐DRC	62	1950	0.032	87	56.3	0.005

lac Pempere	1	18.9	0.053	1	0.8	0.005

Tshangalele	6	362.5	0.017	12	5.5	0.004

Tanganyika‐DRC	256	14,800	0.017	458	243.6	0.004

Kisangolungwe	10	326	0.031	10	8.3	0.003

Albert‐DRC	34	2420	0.014	63	31.6	0.003

Lualaba	4	226	0.018	6	3.4	0.003

Kivu‐DRC	21	1370	0.015	33	19.5	0.003

Mbula‐Matari	12	659	0.018	12	11	0.002

de N'Zilo	1	200	0.005	1	0.9	0.001

Mobutu	1	5300	0	1	0.9	0

Mocro	1	15,100	0	1	1	0
Egypt	Timsah	20	14	1.429	41	15.7	0.304
	Idku	14	62.78	0.223	21	10	0.031
	Bitter Lake	9	250	0.036	21	7.5	0.009
	Mariout	7	106.1	0.066	9	4.7	0.008
	Tismah	1	14	0.071	1	0.6	0.006
	Karun	10	233	0.043	14	7.4	0.006
	Manzala	42	1360	0.031	61	33.9	0.005
	Burrullus	11	462	0.024	17	7.5	0.003
	Waadi El‐Raiyan Lakes	1	113	0.009	1	0.6	0.001
	Zietoon	1	120	0.008	1	0.5	0.001
	Nasser	9	4357	0.002	9	7.2	0
Ethiopia	Afrera	2	100	0.02	8	1.8	0.009
	Awasa	5	129	0.039	9	3.3	0.007
	Ziway	12	440	0.027	23	8.9	0.005
	Tana	27	2156	0.013	84	22.5	0.004
	Hayq	1	23	0.043	1	0.7	0.004
	Chamo	4	317	0.013	8	3.1	0.002
	Abaya	8	1162	0.007	16	6	0.001
	Ganjule	4	328	0.012	4	2.5	0.001
	Langano	2	230	0.009	2	1.1	0.001
	Turkana‐ET	3	1430	0.002	3	2.7	0
Gabon	Nzile	21	4.66	4.506	29	9.7	0.322
	Ndeguelie	20	7.21	2.774	28	8.4	0.185
	Kebanda	12	3.25	3.692	20	2.4	0.181
	Ingoyo	22	11.3	1.947	30	10.3	0.145
	Menguegne	13	4.14	3.14	17	3.9	0.119
	Ayem	20	12.4	1.613	28	8.4	0.107
	Nkonié	19	22.4	0.848	35	7.3	0.082
	Ezanga	24	68.5	0.35	36	13.3	0.034

Avanga	13	31.2	0.417	21	4.2	0.026

Azingo	19	58.5	0.325	31	6.6	0.024

Anengue	20	88	0.227	32	8.3	0.018

Onangue	46	288	0.16	62	29	0.016
Ghana	Bosomtwe	12	49	0.245	20	7.5	0.034
	Volta	64	8502	0.008	71	46.3	0.001
	Aby Lagoon	1	424	0.002	1	0.8	0
	Volta lake	1	8502	0	1	0.5	0
Kenya	Sare	8	5	1.6	22	6.2	0.427
	Chala‐KE	2	2.6	0.769	6	1.8	0.262
	Kanyaboli	12	15	0.8	28	8.9	0.17
	Jipe‐KE	4	24	0.167	12	3.3	0.055
	Nakuru	3	52	0.058	12	2.3	0.021
	Magadi	4	100	0.04	9	3.1	0.009
	Victoria‐KE	108	4100	0.026	308	95.5	0.008
	Naivasha	3	150	0.02	11	2.3	0.007
	Baringo	7	168	0.042	7	5.7	0.004
	Turkana‐KE	47	6140	0.008	69	41.6	0.001
	Jilore	2	444	0.005	6	1.5	0.001
Liberia	Piso	14	103	0.136	26	0	0
Madagascar	Kinkony	16	100	0.16	38	12.7	0.038
	Itasy	7	35	0.2	11	5.3	0.027
	Alaotra	7	900	0.008	21	5.2	0.002
Malawi	Chiuta	36	199	0.181	53	18.8	0.018
	Malombe	32	450	0.071	45	20.2	0.008
	Nyasa‐MW	412	24,400	0.017	760	326.5	0.003
	Chilwa	16	600	0.027	22	6	0.002
	Kingiri	1	0.29	3.448	1	0	0
Mali	Faguibine	11	590	0.019	11	0	0
Mozambique	Machane	5	3.25	1.538	11	2.8	0.248
	Piti	27	27.3	0.989	56	21.5	0.211
	Chualo	16	13.3	1.203	20	13.4	0.16
	Chingute	8	9.48	0.844	14	5.3	0.117
	Pave	6	9.49	0.632	8	3.6	0.057
	Chicunga	6	26.3	0.228	9	4.6	0.035
	Chicamba	15	160	0.094	17	11.5	0.01
	Poelela	5	113	0.044	7	3.5	0.005
	Nyasa‐MZ	140	6400	0.022	198	126.6	0.004
	Cabora‐Bassa	2	2739	0.001	2	1.8	0
Namibia	Liambezi	31	1798	0.017	33	22.5	0.002
	Nyasa	43	29,600	0.001	76	32.3	0
Nigeria	Toidi	7	0.93	7.527	7	2.8	0.37
	Kainji	57	1243	0.046	62	35	0.004
Rwanda	Rukira	2	0.5	4	2	1.6	0.41
	Mpanga	21	9.5	2.211	28	16	0.272
	Kilimbi	5	3.47	1.441	9	2.8	0.183
	Birengero	4	3.16	1.266	6	2.8	0.179
	Sake	12	14.3	0.839	23	8.8	0.149
	Bilira	5	5.4	0.926	9	3	0.122
	Rumira	3	2.2	1.364	3	2	0.114
	Mirayi	1	3.75	0.267	5	0.6	0.093
	Hago	12	16.1	0.745	16	8.8	0.085
	Rwanyakizinga	14	23.6	0.593	22	10.2	0.083
	Rweru‐RW	9	20	0.45	14	6	0.057
	Mugesera	10	39	0.256	23	7.2	0.056
	Ihema	28	86	0.326	44	21.9	0.051
	Karago	1	1.18	0.847	1	0.5	0.051
	Lokondo	2	26.1	0.077	10	1.8	0.044
	Luhondo	3	26.1	0.115	10	2.3	0.042
	Gashanga	1	2.1	0.476	1	0.6	0.036
	Mohasi	10	34.1	0.293	12	7.6	0.034
	Cyohoha‐RW	4	19	0.211	4	2.6	0.017
	Bulera	2	54	0.037	5	1.4	0.01
	Kivu‐RW	41	1000	0.041	62	34.9	0.007
	Lohondo	1	26.1	0.038	1	0.8	0.004
	Mutanda	1	22	0.045	1	0.5	0.003
	Chahafi	1	26.6	0.038	1	0.5	0.002
Senegal	Guiers	58	170	0.341	62	0	0
Sierra Leone	Mabesi	1	23.1	0.043	1	0	0
South Africa	Cubhu	13	4.62	2.814	27	7.8	0.419
	Mpungwini	11	2.67	4.12	11	8.9	0.416
	Mzingazi	19	11	1.727	45	12.2	0.349
	Teza	8	2.62	3.053	14	3.7	0.297
	Jeffreys Bay	29	20.79	1.395	43	26.9	0.24
	Nhlabane	7	5.86	1.195	10	3.9	0.11
	Kuhlange	15	31.6	0.475	28	11.4	0.08
	Sibaya	27	64	0.422	51	18.9	0.07
	St Lucia Lake	68	350	0.194	142	57.7	0.043
	Funduzi	6	15	0.4	6	3.9	0.032
	Mentz	9	34.52	0.261	12	6.3	0.028
	Chrissie	3	18	0.167	3	2.1	0.014
South Sudan	Yirol	11	7.13	1.543	11	5.3	0.094
	Shambe	10	30.3	0.33	14	3.3	0.025
	No	25	100	0.25	29	13.3	0.02
Sudan	Nubia	11	892	0.012	11	0	0
Tanzania	Lutamba	3	1.7	1.765	3	2.2	0.164
	Jipe‐TZ	4	15	0.267	16	3.2	0.103
	Ilamba	2	8.1	0.247	6	1.7	0.083
	Igombe	3	11.7	0.256	7	2.2	0.049
	Chidya	2	7	0.286	2	1.4	0.025
	Kitere	2	7.9	0.253	2	1.3	0.02
	Nyamagoma	8	108	0.074	12	6.7	0.012
	Kitangiri	2	105	0.019	9	1.4	0.007
	Babati	1	21	0.048	1	1	0.006
	Burigi	1	70	0.014	5	0.6	0.005
	Tanganyika‐TZ	272	13,500	0.02	480	257.2	0.004
	Nyasa‐TZ	104	5569	0.019	177	98.8	0.004
	Sagara	5	362	0.014	13	3.6	0.003
	Manyara	5	470	0.011	12	4.1	0.003
	Natron	6	1040	0.006	19	5.4	0.002
	Victoria‐TZ	124	33,700	0.004	428	116.9	0.002
	Rukwa	39	5760	0.007	60	35.2	0.001
	Eyasi	4	1050	0.004	11	2.7	0.001
	Sulunga	2	1029	0.002	5	1.5	0
Togo	Togo	7	64	0.109	7	0	0
Tunisia	Sebhika Kelbia	2	124	0.016	2	0	0
Uganda	Nakabale	10	7	1.429	38	5.9	0.458

Dalaja	2	1.7	1.176	6	1.2	0.345

Nabugabo	42	24	1.75	76	31.3	0.311

Lemwa	14	10	1.4	38	7.5	0.286

Nakuwa	16	8	2	26	9.7	0.25

Opeta	40	42	0.952	100	28.1	0.22

Nyaguo	27	33	0.818	65	17.7	0.172

Mburo	12	10.4	1.154	22	7	0.165

Owapet	6	5.23	1.147	10	3.4	0.147

Nabisojo	7	6	1.167	13	3.2	0.131

Nakivale	8	26	0.308	18	5.5	0.067

Meito	4	14.64	0.273	10	2.2	0.052

Nyamusigire	4	4.4	0.909	4	1.7	0.049

Kachiira	13	36.3	0.358	21	7.9	0.048

George	56	250	0.224	92	46.8	0.04

Saka	1	1.1	0.909	1	0.3	0.038

Bisina	45	349.31	0.129	118	32.1	0.032

Kijanebolola	9	42	0.214	17	5.4	0.031

Edward‐UG	43	675	0.064	107	36.1	0.018

Bunyonyi	4	57	0.07	8	2.7	0.014

Kwania	31	508	0.061	71	21.8	0.013

Kyoga	58	1821.6	0.032	131	45.3	0.007

Albert‐UG	62	2850	0.022	123	54.1	0.005

Wamala	10	187	0.053	12	5	0.004

Victoria‐UG	177	31,000	0.006	609	158.7	0.002
Zambia	Chila	7	1	7	7	3.3	0.406
	Wakawaka	10	4.59	2.179	10	5.3	0.143
	Ishiba	7	7.5	0.933	11	3.3	0.113
	Tanganyika‐ZM	213	2000	0.107	347	179.4	0.019
	Mweru‐ZM	81	2700	0.03	106	58.5	0.004
	Kariba‐ZM	50	2700	0.019	76	35.3	0.003
	Bangweulu	68	15,100	0.005	100	47.3	0.001
Zimbabwe	Kyle	5	249	0.02	9	2.8	0.003
	Kariba‐ZW	52	2700	0.019	82	37.8	0.003
	Chivero	2	26.32	0.076	2	0.5	0.002
	Mutirikwi	1	90	0.011	1	0	0
